# Commensal microbiota in the digestive tract: a review of its roles in carcinogenesis and radiotherapy

**DOI:** 10.20892/j.issn.2095-3941.2020.0476

**Published:** 2022-01-15

**Authors:** Jiali Dong, Yuan Li, Huiwen Xiao, Ming Cui, Saijun Fan

**Affiliations:** 1 Tianjin Key Laboratory of Radiation Medicine and Molecular Nuclear Medicine, Institute of Radiation Medicine, Chinese Academy of Medical Sciences and Peking Union Medical College, Tianjin 300192, China; 2 Department of Microbiology, College of Life Sciences, Nankai University, Tianjin 300071, China

**Keywords:** Oral microbiome, gut microbiome, cancer, radiotherapy

## Abstract

The human microflora is a complex ecosystem composed of diverse microorganisms mainly distributed in the epidermal and mucosal habitats of the entire body, including the mouth, lung, intestines, skin, and vagina. These microbial communities are involved in many essential functions, such as metabolism, immunity, host nutrition, and diseases. Recent studies have focused on the microbiota associated with cancers, particularly the oral and intestinal microbiota. Radiotherapy, the most effective cytotoxic modality available for solid tumors, contributes to the treatment of cancer patients. Mounting evidence supports that the microbiota plays pivotal roles in the efficacy and prognosis of tumor radiotherapy. Here, we review current research on the microbiota and cancer development, and describe knowledge gaps in the study of radiotherapy and the microbiota. Better understanding of the effects of the microbiome in tumorigenesis and radiotherapy will shed light on future novel prevention and treatment strategies based on modulating the microbiome in cancer patients.

## Introduction

The human microbiota, including bacteria, fungi, and viruses, is widely present on the epithelial barrier surfaces of the human body^1^. After a long period of coevolution, commensal microorganisms act as “friends” or “safeguards” that maintain human physiological processes; however, some microbes act as “evildoers” involved in the development of various diseases, including neurologic diseases, metabolic disorders, cardiovascular disorders, infectious diseases, and gastrointestinal complications^2–6^. Cancer morbidity and mortality rank second among all noncommunicable diseases. Through next-generation sequencing technologies, research on the roles of the microbiome, particularly those in the digestive tract, in cancer has experienced a renaissance, and the understanding of the configurations of human flora in tumor development and treatment has gradually been clarified^7–13^. Microbes appear to be involved in approximately 20% of human cancers, and the relationship between cancer and the microbiome is complex^14,15^. Microbes not only promote tumorigenesis but also affect the efficacy and prognosis of therapies^11,16^. For instance, previous studies have indicated that total abdominal irradiation influences gut microbiota configurations, and gut flora and the derived metabolites can be used as novel remedies to protect against radiation-induced toxicity to the hematopoietic system and gastrointestinal tract^17–19^. Currently, the symbiotic microbiome has become a hotspot of research in tumor occurrence and therapy.

In this review, we provide an overview of the relationship between commensal microbiota and carcinogenesis, with a focus on the effects of digestive tract microbiota on radiotherapy. Importantly, modulation of oral or intestinal microorganisms might be a potential auxiliary method in tumor radiotherapy.

## Overview of the oral microbiome and cancer development

The oral microflora, the microbial community existing in the human oral cavity, comprises more than 700 bacterial species that are crucial players in resisting pathogens and maintaining oral homeostasis^20^. However, the oral microbiota is also responsible for a variety of oral diseases, including periodontal diseases, dental caries, and oral cancer^21,22^. Increasing evidence suggests that oral microorganisms are associated with carcinogenesis in distant organs, such as colorectal cancer, esophageal cancer, and pancreatic cancer^23^.

### Head and neck tumors

High-throughput sequencing has shed light on the complex functions of microorganisms, thus providing an in-depth understanding of the mechanisms underlying head and neck cancers. Head and neck cancers are malignancies that mainly arise in the oral cavity, hypopharynx, oropharynx, and larynx^24^. Periodontitis, poor oral hygiene, and oral flora imbalance are potential risk factors for the development of head and neck squamous cell carcinoma, primarily oral squamous cell carcinoma (OSCC) and oropharyngeal squamous cell carcinoma (OPSCC)^24,25^. Smoking and excessive drinking are considered “time bombs” that negatively affect health. In OSCC, in the presence of other known risk factors, tobacco and alcohol consumption disrupt the oral microbiota, thus resulting in oral cancer development^26^. The oral microbiota converts alcohol into acetaldehyde, a mutagen, thereby promoting carcinogenesis of the head and neck mucosa^24,27,28^.

A prototypic example of a condition associated with bacterial infection is human papilloma virus (HPV), an independent risk factor for OPSCC, which induces the overexpression of the protein P16 and the oncogenes E6 and E7, thereby driving carcinogenesis^29–31^. People with HPV infection also have elevated abundance of the genera *Gemellaceae* and *Leuconostoc*^32^. In addition, *Porphyromonas gingivalis* (*P. gingivalis*) and *Fusobacterium nucleatum* (*F. nucleatum*) infection activate IL-6/STAT3 signaling, spur periodontitis, and participate in the pathogenesis of OSCC^12,33^. To date, substantial evidence on how periodontal pathogens induce OSCC is lacking. Further studies, particularly clinical studies, are warranted to improve knowledge of cancer-provoking microbial characteristics.

Accompanying the insights into the pathogenic factors in oral cancers, mounting and compelling evidence indicates that patients with cancer have a distinctive oral microbiome compared with that in healthy controls. The oral epithelium in patients with head and neck squamous cell carcinoma has a lower abundance and diversity of microbiota^32^, thus validating that an oral flora imbalance promotes the development of tumors. In detail, species of some salivary bacterial genera, such as *Prevotella melaninogenica* (*P. melaninogenica*), *Capnocytophaga gingivalis* (*C. gingivalis*), and *Streptococcus mitis* (*S. mitis*), are correlated with OSCC^34^. In addition, dysbiosis at tumor sites may show a signature of markedly decreased abundance of the phyla *Firmicutes* and *Actinobacteria* compared with that in paired normal tissue^35,36^. Previous studies have revealed only a correlation between some special microbes and oral tumors; however, the complexity is less well characterized and requires further investigation. Such research cannot be limited to the study of a single pathogen^37^.

### Colorectal cancer

The digestive tract is a tubular passage typically extending from the mouth to the anus or cloaca. Microorganisms in the upper digestive tract may migrate to and colonize the lower digestive tract. In colorectal cancer (CRC), some oral bacteria emerge in the lower digestive tract, particularly at tumor sites. For example, periodontal pathogens including *Fusobacterium* and *Porphyromonas* are detectable in samples from patients with CRC. Other oral pathogens, such as *Treponema denticola* and *Prevotella intermedia*, are also associated with an increased risk of CRC^38–41^. In addition, *Fusobacterium*, particularly *F. nucleatum*, is a genus frequently observed in CRC^42,43^. Colon ectopic *F. nucleatum* is a CRC-promoting bacterium, and scientists have further explored the mechanism underlying how *F. nucleatum* promotes CRC carcinogenesis, including immune modulation, virulence factors, microRNAs, and bacterial metabolism^44^. In addition to *Fusobacterium*, orally originating microorganisms, such as *Gemella*, *Peptostreptococcus*, and *Parvimonas*, are found in the lower digestive tract and are associated with the progression of CRC^40^; however, additional studies are needed to explore the virulence factors and carcinogenic potential of these genera.

### Pancreatic cancer

Multiple publications have demonstrated an association between increased pancreatic cancer risk and poor oral health^45,46^. Oral health is strongly affected by the activities of oral flora, and scientists have hypothesized that the bacterial conditions in the oral cavity may be associated with a high pancreatic cancer risk^47,48^. In many of these studies, a higher frequency of the genera *Bacteroides* and *Granulicatella adiacens* is more common in patients with pancreatic cancer than in healthy people; however, some bacteria, including *Neisseria elongata* and *S. mitis*, are present at lower concentrations^49,50^. A previous study has confirmed that elevated blood serum antibodies to *P. gingivalis*, an oral pathogen, may contribute to a higher risk of pancreatic cancer^51^. Given this evidence, researchers have compared oral bacterial samples between pancreatic cancer patients and healthy controls, and found that the presence of *Aggregatibacter actinomycetemcomitans and P. gingivalis* in the oral cavity is associated with pancreatic carcinogenesis, whereas the phylum *Fusobacteria* and the genus *Leptotrichia* are protective and decrease the risk^13^. Although dysbiosis of the oral microbiota has been implicated in pancreatic cancer^13,49^, direct evidence defining a causal relationship between oral dysbiosis and early distal cancer is lacking. However, in light of the clear relationship between periodontal pathogens and pancreatic cancer, the therapeutic or prophylactic implications in preventing periodontal pathogens might potentially decrease the risk of pancreatic cancer.

## Gut microbiome and cancer development

The human intestinal flora consists of more than 1,000 types of bacterial species, and the gut exists in homeostasis in healthy individuals through interactions between the host and microbiome, thus preventing the invasion of pathogens^52^. However, intestinal dysbiosis leads to unfavorable host effects, such as tumorigenesis^53^. The mechanisms through which the gut microbiome affects tumor development are diverse, including the creation of a local chronic inflammatory state, as well as genotoxic effects^27,54^. Here, we summarize available studies and characterize the gut microflora features in human cancers.

### Colorectal cancer

Similarly to other diseases, CRC results from various multifactorial genetic factors and environmental stimuli^55,56^. Genetic predisposition syndromes for CRC account for a minority of cases^57^. Family studies have shown that heritability as the etiological agent for CRC accounts for only 12%–35% of the total incidence^55,58^, thus suggesting that environmental factors play crucial roles in CRC development. The roles of the commensal microbiome in CRC, particularly the microbiota dwelling in the intestine, are increasingly being recognized.

As early as the 1960s, scientists studied the effects of intestinal microflora on the CRC susceptibility in mouse models^59^. Later evidence identified the relationship between gut flora dysbiosis and colorectal tumorigenesis, and most studies indicate that shifts in the gut microbiome composition contribute to CRC development^60^. A significant discrepancy in microbiota configuration between the tumor site and peritumoral milieu has been reported^61,62^. Fecal particles carry substantial physiological and pathological information and have the potential to be used for noninvasive diagnosis. Focusing on fecal samples from patients with CRC, researchers have discovered that microbiota diversity and *Clostridia* frequency are lower than those in the non-CRC population, whereas the abundance of *Porphyromonas* and *Fusobacterium* at the genus level is higher^42^. Wong and colleagues^63^ have collected and transplanted the stool microbiome from patients with CRC to experimental animals, and found that the patients’ fecal flora promoted colorectal carcinogenesis in mice, thus suggesting that irregular gut microbiota might be a driver of CRC. The mechanisms through which gut dysbiosis promotes CRC are presumed to involve disorders in host metabolism and mucosal immune responses induced by microorganisms^64,65^. High-throughput sequencing has allowed researchers to identify specific gut pathogens positively associated with CRC. For instance, infection with *Streptococcus bovis* (*S. bovis*) has been determined to indicate a high risk of colonic tumors^66^, and enterotoxigenic *Bacteroides fragilis* (ETBF) is significantly enriched in patients with CRC compared with the healthy population. Several of the malgenic mechanisms, including the production of reactive oxygen species that induce DNA damage and the activation of pathways involving the proinflammatory cytokine IL-17 and Wnt, are closely associated with the induction of CRC by ETBF and have been comprehensively reviewed^67–69^. Alternatively, chronic inflammation targets the intestinal microbiota, disturbs the metabolome, and produces metabolites with direct genotoxicity. For example, pks+ *Escherichia coli* (*E. coli*) drives tumorigenesis by inducing DNA mutagens^70^, and *Campylobacter jejuni* promotes CRC occurrence through the direct genotoxic action of cytolethal distending toxin^71^.

### Liver cancer

Specific enteric microbes and microbial dysbiosis affect the status of distant organs in a sophisticated and orchestrated manner. For example, gut microbiota-derived heterogeneous metabolites enter the blood circulation and affect the physiological and pathological processes of nonenteric tissues such as the liver. A gut flora imbalance potentiates hepatocellular carcinoma by damaging DNA, activating oncogenic signaling pathways, and releasing tumor-promoting compounds^72,73^. The intestine and the liver are anatomically and physiologically linked *via* the portal vein, thus forming the “intestinal-liver axis”^74–76^, and the gut microbiome is a critical factor mediating this axis^75^. Intestinal dysbiosis and increased intestinal permeability facilitate the transfer of microorganisms from the intestine to the liver, including gut microbial components called microbe-associated molecular patterns, metabolites, and the microbiota itself. These elements cause an inflammatory response and may potentially lead directly to carcinogenesis^77,78^. Bile acids (BAs) are active biocomponents that regulate the metabolic pathways of hepatocytes and intestinal epithelial cells. However, enteric microorganisms modify primary BAs into secondary BAs such as deoxycholic acid, thereby leading to DNA damage, hepatotoxicity, and carcinogenesis^79,80^. Furthermore, the production of BAs may alter immune function, for example, through natural killer T cells’ influencing tumor growth^81^. Gram-negative or gram-positive-bacteria-derived lipopolysaccharide or lipoteichoic acid interacts with Toll-like receptor (TLR) 4 or TLR2 and increases inflammatory status through the innate immune response, thus promoting liver fibrosis and cancer^82–84^. Additionally, the gut microbiota is associated with obesity, infectious hepatitis, and nonalcoholic steatohepatitis, all of which may lead to cirrhosis and consequently hepatocellular carcinoma^72^.

### Breast cancer

Given the lack of substantial evidence, integrating the gut microbiota into an understanding of breast cancer occurrence is difficult. However, studies have shown that the intestinal flora in patients with breast cancer is different from that in healthy controls^85^, and mounting studies indicate that the gut microbiota is involved in the metabolism of estrogen, which promotes carcinogenesis. The possible mechanisms through which the gut microbiota affects breast cancer include regulating circulating estrogen and phytoestrogen profiles, interfering with energy metabolism and obesity, and impeding antitumor immune function^86,87^. Strikingly, recent studies have shown that deletion of the gut microbiome by using an antibiotic cocktail increases breast cancer cell metastasis in mouse models, thus highlighting the importance of gut microbes in breast cancer^88^. Given the success of preliminary outcomes, further preclinical, clinical, and epidemiological studies are urgently needed to elucidate the relationship between the gut microbiome and breast cancer.

## The microbiome and cancer radiotherapy

**Figure 1:**
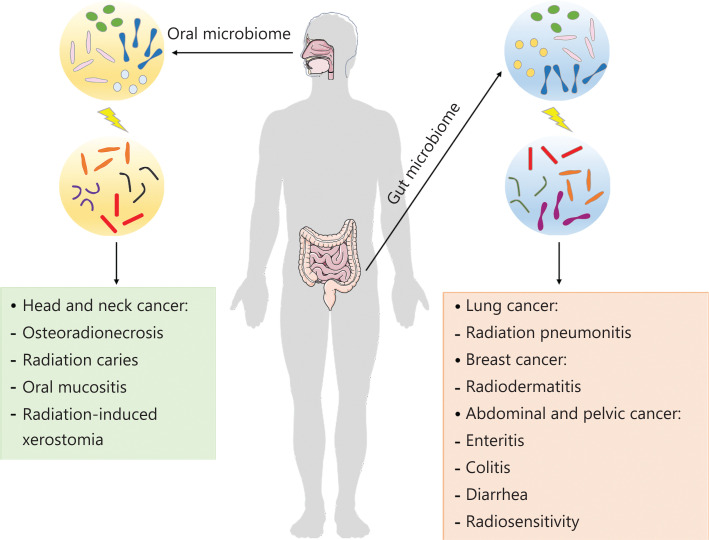
The influence of the microbiota on cancer radiotherapy. Oral microbes contribute to radiation-induced chronic complications including osteoradionecrosis, caries, oral mucositis, and xerostomia in head and neck tumors. Gut microbes can influence the adverse effects and efficacy of radiotherapy for various cancers.

Radiotherapy is one of the major remedies for cancer and is regarded as a milestone for oncotherapy^89^. Approximately 50% of patients newly diagnosed with cancer and many patients with recurrent or persistent tumors undergo radical or palliative radiotherapy with the explicit goal of reducing tumor growth and inhibiting tumor metastasis^90^. Compelling evidence suggests that the microbiome is strongly affected by radiotherapy (**Figure 1**), including efficacy, prognosis, and adverse effects^91,92^.

### The microbiome in radiotherapy for head and neck tumors

Radiotherapy is the first-line option for head and neck cancers (HNCs). Although surgery is the oldest and most traditional treatment for HNC, modern radiotherapy has been demonstrated to be on par with surgery in treating HNC, particularly when lesions are in early stages. Iatrogenic irradiation of the head and neck region is inevitably associated with deleterious effects to many normal organs and precipitates irreversible oral complications, such as osteoradionecrosis^93^, radiation caries^94^, mucositis^95,96^, and low saliva^97^, thus decreasing patient quality of life. The human oral cavity is home to a wide range of microbiota, with more than 700 species of microorganisms identified to date^98,99^. In patients receiving radiation therapy, the dynamic and stable oral ecosystem is undermined. In 1975, Brown and colleagues^100^ found that radiotherapy-reduced saliva flow is accompanied by pronounced shifts in some microbial populations, thus linking radiation exposure to the oral microbiota for the first time. Subsequently, studies on oral bacteria and radiotherapy have gradually become a novel research focus in studies on HNC. For example, studies on the oral microbial changes have demonstrated that radiotherapy increases the *Candida* frequency in the oral cavity in patients with squamous cell carcinoma^101^. At 6–8 months after radiotherapy, the abundance of specific microflora, such as *Candida albicans*, *Enterococci*, *Lactobacillus* spp., and *Streptococcus mutans*, are considerably higher than those in patients without radiotherapy^102^. Saliva serves as an essential safeguard in the oral cavity that helps maintain a “healthy mouth”; for example, saliva flow reduction is an emblematic “red flag” suggesting dental caries. Saliva lubricates oral surfaces and maintains the structural integrity of teeth by attenuating demineralization, clearing food, facilitating remineralization, and bolstering the adaptive and innate arms of the host defenses. More recently, exposure to 20 Gy has been found to decrease salivary function by as much as 80%^103^. Owing to the high radiosensitivity of salivary glands, iatrogenic irradiation of the head and neck region harms salivary glands and retards saliva secretion^104^. Saliva consists of a spectrum of primary nutrients for the resident oral microbiota; thus, the radiotherapy-induced decrease in saliva impairs the equilibrium of the oral microbiota. In addition, prolonged hyposalivation and xerostomia significantly affect the microflora environment, thus further influencing the oral microbiota configurations^105^.

Before next-generation sequencing technologies, the traditional methods to assess microorganisms included screening and culturing any microbes or target microbes on special culture media. The emergence of next-generation sequencing technologies has enabled discovery of the diversity of microbial gene repertoires^8^, thus enabling further study of how radiotherapy affects oral microorganisms. Previous studies have reported that a total of 140 species of bacteria belonging to 13 phyla are found in the dental plaques in patients with head and neck tumors before and during radiotherapy, and a negative relationship exists between the number of OTUs and radiation dose^106,107^. Beyond research focusing on the changes in microorganisms, studies are increasingly demonstrating the important roles of oral microbes in the pathogenesis of radiation-induced chronic long-term complications, including caries and oral mucositis. For example, Mougeot and colleagues^108^ have reported a potential protective role of *Abiotrophia defectiva* (*A. defectiva*) and a cariogenic role of *Prevotella melaninogenica* (*P. melaninogenica*), on the basis of analysis of the oral microbiome profiles of patients with HNC. However, whether the oral microbiota can be used as a biomarker to predict the incidence and severity of oral toxicity remains uncertain. Several studies have demonstrated the emergence of pathogenic bacteria associated with mucositis in patients with HNC during radiotherapy, thus suggesting that oral microbes may contribute to the onset and severity of oral mucositis^109,110^. Emerging data describe the dynamic variation in oral microbiota during radiotherapy and its association with the incidence and severity of oral mucositis in patients with HNC ^111^. The results show that several bacteria, including *Haemophilus*, *Fusobacterium*, *Tannerella*, *Porphyromonas*, and *Eikenella*, contribute to patient susceptibility to oral mucositis. Some bacteria can also serve as probiotics that mitigate radiation-induced oral injuries. For instance, *Lactobacillus brevis* CD2 lozenges have been suggested to be a safe and effective treatment that decreases the incidence of mucositis in patients with HNC during radiotherapy^112^. Furthermore, increasing attention is being paid to the intriguing therapeutic possibility of regulating salivary microbiota for oral mucositis induced by radiotherapy^113^. One study has shown that a probiotic combination significantly ameliorates radiation-induced oral mucositis by improving the immune response^114^. However, further studies are needed to discover the effects of specific bacterial strains on oral toxicity mediated by radiation therapy for HNC and to provide mechanistic insight.

### The microbiome in radiotherapy for lung cancer and breast cancer

Emerging evidence indicates that the lung has a microbiome of its own. Sputum samples from patients with lung cancer, compared with those from non-lung cancer patients, have higher relative abundance of isolated *Granulicatella*, *Abiotrophia*, and *Streptococcus*^115^. Furthermore, the microbiota in lung tumor tissues shows higher taxa-taxa interactions and lower species richness^116^. One recent study has reported that patients with periodontal disease are at increased risk of developing lung carcinoma, but the biological mechanisms remain undetermined, and whether oral microbes play a key role in tumorigenesis requires further detailed study^117^. However, the enteric counterparts of the oral flora and gut microbiome have been demonstrated to have prognostic value for lung cancer patients^118,119^. Despite the above evidence linking the microbiota to lung cancer, the role of local (lung) or distal (oral/gut) microbiota in lung cancer has not been clarified. In terms of microflora and lung cancer radiotherapy, a recent study has provided evidence that the intestinal microbiota protects against radiation pneumonitis, as demonstrated through fecal microbiome transplantation into irradiated mice; therefore, the gut-lung axis may be an innovative therapeutic avenue for protecting against radiation-induced lung injury^120^. Nevertheless, the protective potential of special gut microbiota in lung cancer during radiotherapy is not yet well defined. Longitudinal epidemiological and prospective studies are needed to determine the effects of microbial communities on lung cancer radiotherapy.

Chan and colleagues^121^ have used 16S rRNA gene amplicon sequencing to explore the effects of the local breast ductal microbiome on breast cancer. The authors have presented the first evidence of a lower incidence of a genus from the family *Sphingomonadaceae* and higher incidence of the genus *Alistipes* in nipple aspirate fluid collected from patients with breast cancer. Additional studies have reported that bacterial species, including those from *Bacillus*, *Enterobacteriaceae*, and *Staphylococcus*, are enriched in breast tissue from patients with breast cancer^122^. Strikingly, recent evidence has further demonstrated a functional relationship between the gut microbiome and breast cancer^88^. For example, a study has indicated that gut microbiome disorder promotes breast cancer metastasis in mouse models, thus suggesting that greater attention should be paid to the association between the gut microbiome and breast cancer^123^. Radiation therapy, a major therapeutic modality in the treatment of breast cancer, can be used in breast conservation and postmastectomy settings. Patients with breast cancer experience acute or chronic dermatitis in response to radiotherapy, consisting of atrophy, fibrosis, or pigmentation alterations, which can be mitigated by topical steroids and skin hygiene^124^. The mechanisms of radiation-induced dermatitis include the activation of T cells and the prevention of epidermal repair by bacterial superantigens, particularly those from *Staphylococcus aureus* (*S. aureus*)^125^. Recently, the microbiome has been widely considered an indirect factor that affects the immune system by shifting the balance of glucose utilization and fatty acid oxidation during radiotherapy^126^. M1 macrophages promote the radiosensitivity of breast cancer cells. In contrast, M2 macrophages cause radioresistance in breast cancer cells, an effect attributed to STAT6 phosphorylation and M2 polarization mediated by IL-4/IL-13^127,128^. To date, a comprehensive understanding of the causal relationship between microbiota and radiotherapy for breast cancer remains lacking.

### The microbiome in radiotherapy for abdominal and pelvic cancer

Radiotherapy is an effective treatment for abdominal and pelvic cancer, on the basis of its genotoxic effect on tumor cells. However, a healthy bowel is inevitably exposed to radiation, thus causing adverse sequelae that manifest as gastrointestinal toxicity^129^. As an organ, the intestine comprises many different cell types, including vascular, enteric immune, and nervous system cells, all of which are influenced by irradiation to various degrees^130^. Radiation exposure damages the intestinal barrier, alters the abundance and composition of gut microbiota, and causes apoptosis in intestinal crypts^17,19,131,132^. The pathogenesis of enteritis, colitis, and diarrhea in mouse models and patients receiving radiation therapy has been found to be associated with a gut flora imbalance, which limits therapy completion^133^. Gut dysbiosis is typically characterized by decreased microbial abundance and/or significant shifts in composition. Evidence suggests that gut microbial dysbiosis may be a biomarker predicting radiation enteropathy. Patients with pelvic radiotherapy-induced diarrhea possess a significantly higher relative *Firmicutes/Bacteroides* ratio as well as differences in the relative abundance of *Clostridia* cluster XVIII^134^. Studies support that *Clostridia* cluster XVIII promotes the expansion and differentiation of T cells, which fight against colitis and allergic diarrhea^135^. Another clinical study has shown that patients receiving pelvic radiotherapy show prominent changes in their gut microbiota composition after radiotherapy, particularly in *Firmicum* and *Fusobacterium*, which have been found to decrease by 10% and increase by 3%, respectively^136^. Evidence from murine models indicates that rectal irradiation can induce local microbial dysbiosis, which in turn increases mucosal IL-1β secretion, thus resulting in radiation-induced colonic damage^137^. The above studies clearly show that the gut microbiota composition is associated with the pathogenesis of radiation-induced damage, but larger studies are needed to confirm these findings.

Our team has long focused on gut microbiota and radiation toxicity, and has demonstrated that gut microbiota configurations relate to radiation injuries in hosts, and that modulation of the intestinal microbiome mitigates hematopoietic system and gastrointestinal tract toxicity after irradiation challenge^17–19,138,139^. Intuitively, certain gut microbiota configurations significantly ameliorate the response and toxicity to acute radiation syndrome^18^. We propose that rehabilitation strategies for patients undergoing radiotherapy for cancer should take the sex of patients into account^17^. In terms of mechanism, our studies of gut microbiota-produced metabolites, including valeric acid and indole 3-propionic acid, provide a new perspective regarding microbiome-based remedies for radioactive diseases^19,139^. Recently, a clinical study has provided the first evidence that fecal microbiota transplantation might be safe and effective in improving intestinal symptoms and mucosal injury in patients with chronic radiation enteritis within a certain period of time^140^. However, the relevance of the biological mechanisms through which fecal microbiota transplantation might serve as a therapeutic mitigating radiation-induced toxicity remains to be investigated.

In addition to their roles in pathogenicity, microorganisms have been demonstrated to play key roles in the response to protect the intestinal mucosa against radiotherapy-induced toxicity. For example, probiotic *Lactobacillus rhamnosus* GG has been shown to prevent radiation-induced injury, by decreasing epithelial injury and improving crypt survival, which depends on TLR-2/MyD88/COX-2 signaling^141^. In some clinical studies, probiotics have been shown to decrease the risk of some severe consequences of radiation therapy, such as diarrhea^133,142^. All the above findings support the possibility that probiotics could be used as adjuvant agents during radiotherapy. However, the findings of a meta-analysis show that current evidence does not indicate beneficial effects of probiotics for the prevention of radiotherapy-induced diarrhea; consequently, research efforts should focus on the specific forms of gastrointestinal toxicity and certain microbial phenotypes to develop targeted microbiota manipulation^143^.

The effects of the intestinal microbiota have also been investigated in the setting of radiotherapy efficacy for abdominal and pelvic cancer. Crawford and Gordon^144^ have found that intestinal flora increases the radiosensitivity of endothelial cells and lymphocytes in the mesenchymal cores of the small intestinal villi in germ-free mice receiving total body irradiation, thus providing new viewpoints regarding the relationship between intestinal flora and radiosensitivity. In addition, perturbations in the circadian rhythm elicit alterations in gut bacterial configurations that worsen radiation-induced injuries, as compared with those in mice housed under 12 h dark/12 h light cycles^138^. Antibiotic-treated mouse models have provided further evidence supporting this association^18^. These studies have been further supplemented by published clinical studies demonstrating that circadian rhythm may affect radiotherapy local control and toxicities^145^. Even if the gut microbiome may be a key player in mitigating radiation therapy-associated complications, the direct effects of the intestinal microbiota on the efficacy of radiotherapy remain to be understood, but might be an important and interesting field in radiomedicine.

## Conclusion

In the past few years, knowledge of the microbiome in cancers has rapidly grown, and the mechanisms of tumorigenesis that are attributable to the microbiota are progressively being understood (summarized in **Table 1**). The microbiome is relevant in cancer development and therapy through many routes, *via* bacterial infection or crucially through bacterial dysbiosis; however, the underlying mechanisms remain poorly understood. Moreover, some challenges regarding how the microbiome affects tumor radiotherapy remain to be solved. In general, insights have been gained into the relationship between the oral/gut microbiome and cancer radiotherapy. Specifically, radiotherapy influences the commensal microbiota of hosts, and conversely, the commensal microbiota affects the efficacy and prognosis of radiotherapy. However, much remains to be learned about the mechanisms underlying these events. Furthermore, with advances in cancer radiotherapy, we must consider the factors modulating commensal flora, such as diet, antibiotic usage, and hygiene management. Only by fully understanding these interactions can we know how to optimally modulate the microbiome to enhance radiotherapy efficiency and limit radiation-induced adverse effects.

**Table 1:** The influence of microbes on cancer development

Malignancy	Methods	Main results	References
Head and neck cancer	Meta-analysis	Oral microbiota converts alcohol into acetaldehyde, thus causing cancer.	^26^
	Case-control study	HPV is widely considered an independent risk factor inducing OPSCC.	^30^
	Murine model	*P. gingivalis* and *F. nucleatum* infection promotes OSCC *via* the IL-6-STAT3 pathway.	^33^
	Case-control study	*P. melaninogenica*, *C. gingivalis*, and *S. mitis* are elevated in the saliva of individuals with OSCC.	^34^
	Case-control study	*Actinomyces* and *Firmicutes* are significantly depleted in tumor tissue relative to normal tissue.	^35^
Colorectal cancer (CRC)	Case-control study	*Fusobacterium* and *Porphyromonas* are detectable in samples from patients with CRC.	^40^
	Case-control study	*Treponema denticola* and *Prevotella intermedia* are associated with increased risk of CRC.	^41^
	Murine model	*F. nucleatum* precipitates CRC carcinogenesis *via* immune modulation, virulence factors, microRNAs, and bacterial metabolism.	^44^
	Case-control study	*Gemella*, *Peptostreptococcus*, and *Parvimonas* are found in CRC.	^40^
	Case-control study	Gut microbial dysbiosis contributes to the development of CRC.	^61^
	Case-control study	Lower relative abundance of *Clostridia* and higher relative abundance of *Porphyromonas* and *Fusobacterium* are found in patients with CRC.	^42^
	Murine model	Stool microbiota from patients with CRC promotes colorectal carcinogenesis in mice.	^63^
	Case-control study	*Streptococcus bovis* is a risk factor for colonic tumors.	^66^
	Murine model	ETBF is highly expressed in patients with CRC compared with healthy people.	^67,68^
	Murine model	*E. coli* induces tumorigenesis through generating DNA mutagens.	^70^
	Murine model	*Campylobacter jejuni* promotes CRC through the genotoxic action of cytolethal distending toxin.	^71^
Pancreatic cancer	Case-control study	The *Bacteroides* genus and *Granulicatella adiacens* are more common in patients with pancreatic cancer than healthy people; however, *Neisseria elongata* and *Streptococcus mitis* are present in lower concentrations in pancreatic cancer.	^49^
	Case-control study	*P. gingivalis* may contribute to a higher risk of pancreatic cancer.	^51^
	Case-control study	*Aggregatibacter actinomycetemcomitans* and *P. gingivalis* in the oral cavity are associated with pancreatic carcinogenesis, whereas the phylum *Fusobacteria* and its genus *Leptotrichia* are protective and decrease the risk.	^13^
Liver cancer	Murine model	Gut bacterial metabolites cause DNA damage and carcinogenesis.	^79^
	Murine model	Gut bacteria-controlled bile acids may alter immune function, thus influencing tumor growth.	^81^
	Murine model	Intestinal microbiota and lipopolysaccharide accelerate hepatocarcinogenesis.	^82^
	Murine model	A gram-positive gut microbial component increases the risk of cancer development through creating a tumor-promoting microenvironment.	^83^
Breast cancer	Case-control study	The intestinal flora in patients with breast cancer is different from that in healthy controls.	^85^
	Murine model	Gut dysbiosis affects mammary tumor dissemination.	^88^
